# Supplementary material for: Existence of Quantum Pharmacology in Sartans: Evidence in Isolated Rabbit Iliac Arteries
Source: Int J Mol Sci. 2023 Dec 16;24(24):17559. doi: 10.3390/ijms242417559 (PMC10744031; doi:10.3390/ijms242417559)
Supplement: Supplementary file 1 [file ijms-24-17559-s001.zip › ijms-2703119-supplementary.pdf]

Supplementary Figures

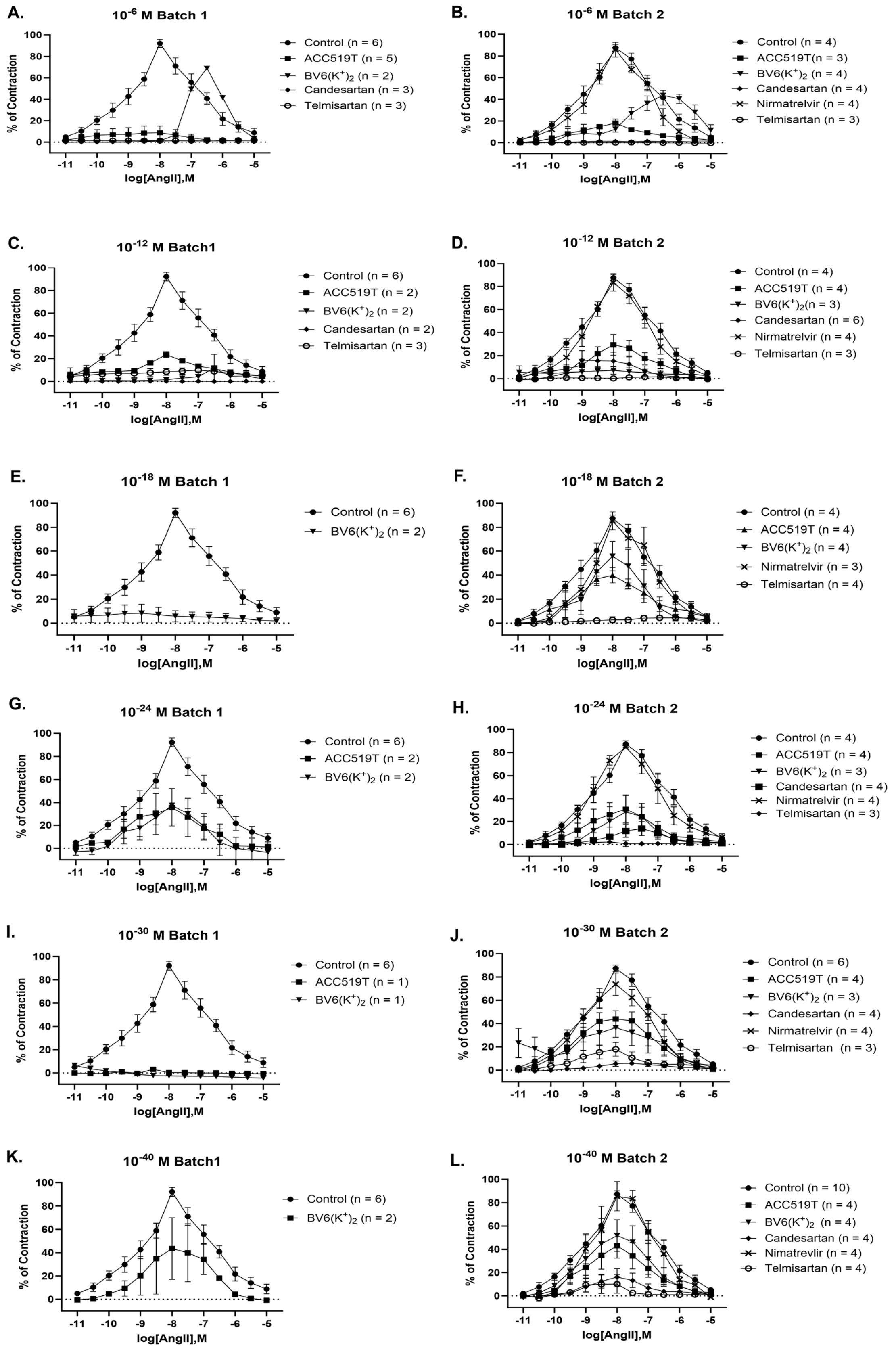

Figure S1. Rabbit batch difference in contraction responses to AngII dose–response effect in isolated iliac arteries pre-treated with various doses of ARBs. Contraction responses of rabbit iliac arteries from two different batches to an AngII dose–response after pre-treatment of with 10^{-6} M in (**A**) batch

1, (**B**) batch 2; 10^{-12} M (**C**) batch 1, (**D**) batch 2; 10^{-18} M (**E**) batch 1, (**F**) batch 2; 10^{-24} M (**G**) batch 1, (**H**) batch 2; 10^{-30} M (**I**), batch 1 (**J**) batch 2; 10^{-40} M (**K**) batch 1, (**L**) batch 2 (mean ± SEM). Abbreviations: ACC519T, benzilimidazole-*N*-biphenyl tetrazole; AngII, angiotensin II; benzilimidazole *bis-N,N′*-biphenyl tetrazole; ARBs, angiotensin receptor blockers; BV6$(K^+)_2$, 4-butyl-N,N0-bis[[20-2Htetrazol-5-yl)biphenyl-4-yl]methyl)imidazolium bromide; SEM, standard error of mean.

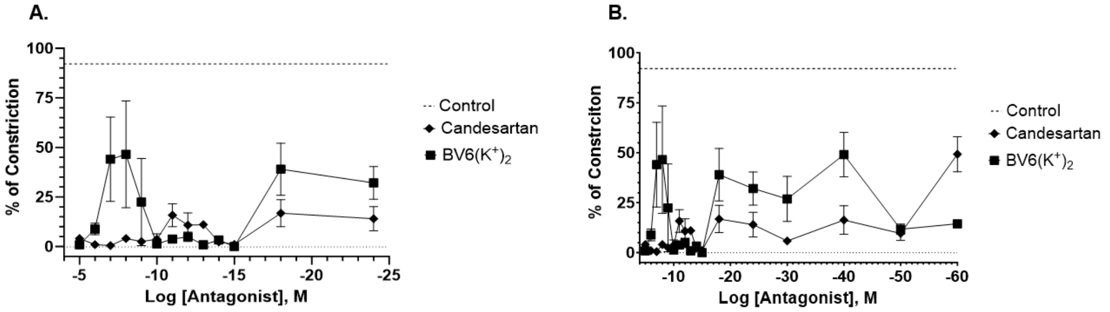

Figure S2: Maximal inhibitory effect of candesartan and BV6$(K^+)_2$ on AngII-mediated contraction. Maximal endpoint AngII contraction responses of antagonist dose–response effect in rabbit iliac after treatment with (**A**) 10^{-5} M to 10^{-15} M, 10^{-18} M, and 10^{-24} M and (**B**) 10^{-5} M to 10^{-15} M, 10^{-18} M, and 10^{-24} M, 10^{-30} M, 10^{-40} M, 10^{-50} M, and 10^{-60} M dose of candesartan, and BV6$(K^+)_2$. Abbreviations: AngII, angiotensin II; BV6$(K^+)_2$, 4-butyl-N,N0-bis[[20-2Htetrazol-5-yl)biphenyl-4-yl]methyl)imidazolium bromide; SEM, standard error of mean.

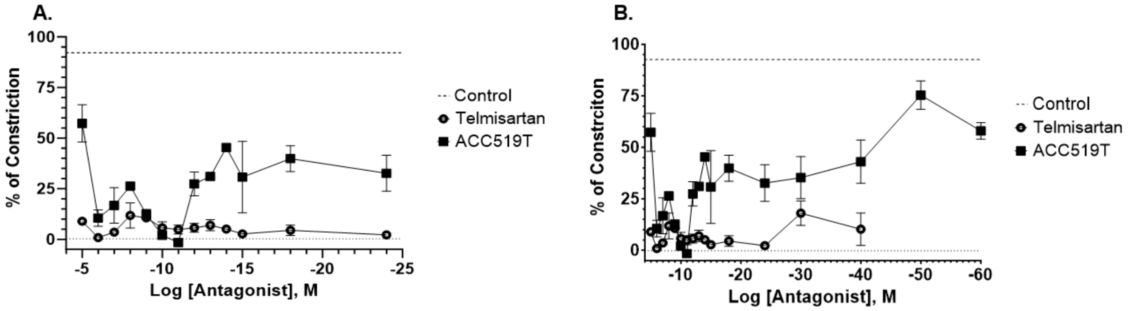

Figure S3. Maximal inhibitory effect of telmisartan and ACC519T on AngII-mediated contraction. Maximal endpoint AngII contraction response of antagonist dose–response effect in rabbit iliac after treatment with (**A**) 10^{-5} M to 10^{-15} M, 10^{-18} M, and 10^{-24} M and (**B**) 10^{-5} M to 10^{-15} M, 10^{-18} M, and 10^{-24} M, 10^{-30} M, 10^{-40} M, 10^{-50} M, and 10^{-60} M doses of telmisartan and ACC519T. Abbreviations: AngII, angiotensin II; ACC519T, benzilimidazole-*N*-biphenyl tetrazole; SEM, standard error of mean.

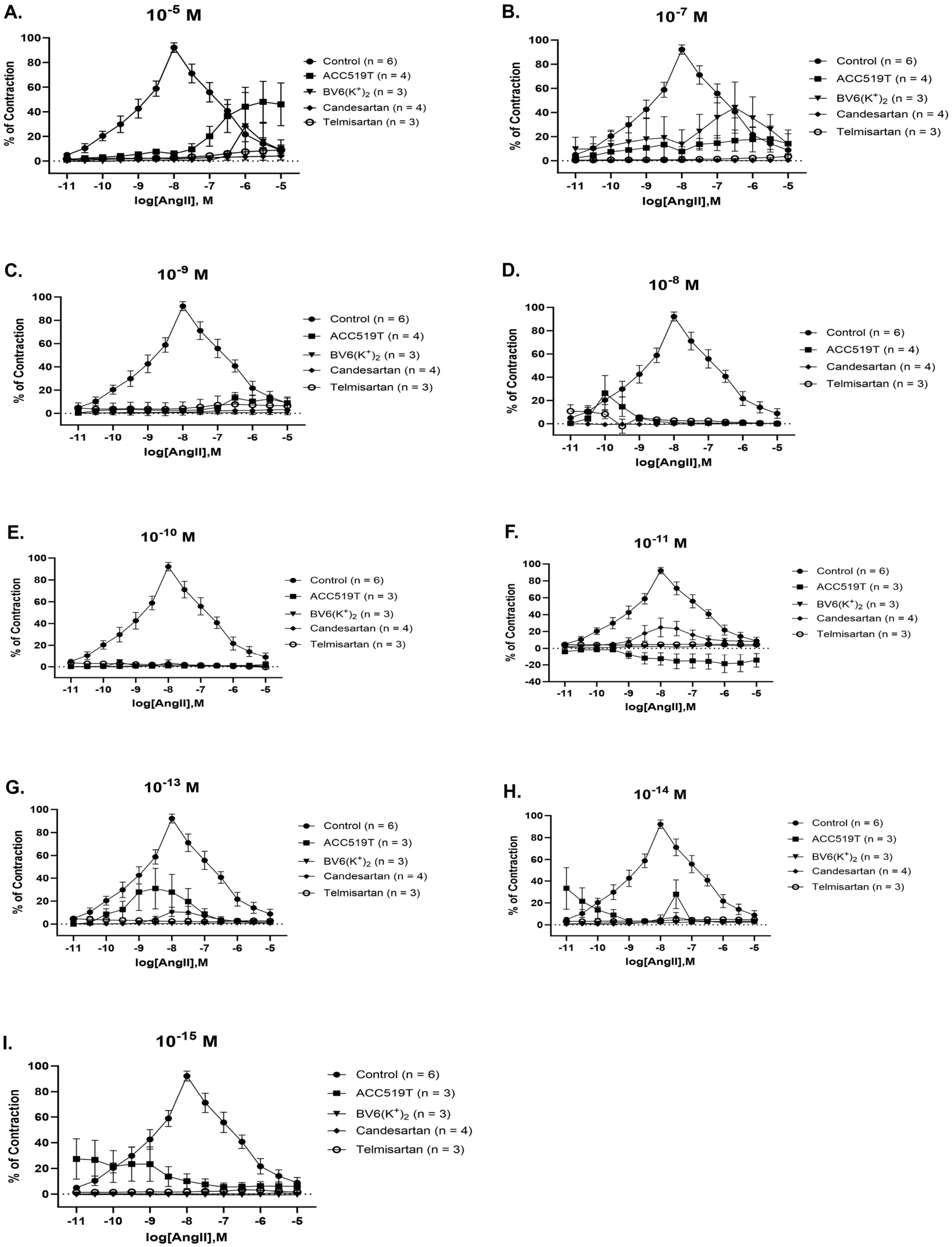

Figure S4. Contraction responses to AngII dose–response effect in isolated rabbit iliac arteries pre-treated with various doses of ARBs. Contraction responses of rabbit iliac to AngII dose–response iliac after pre-treatment with (**A**) 10^{-5} M, (**B**) 10^{-7} M, (**C**) 10^{-8} M, (**D**) 10^{-9} M, (**E**) 10^{-10} M, (**F**)10^{-11} M, (**G**)

10^{-13} M, (**H**) 10^{-14} M and (**I**) 10^{-15} M dose of ACC519T, BV6(K^+)$_2$, candesartan, nirmatrelvir and telmisartan (mean ± SEM) (significance shown in Table 2). Abbreviations: ACC519T, benzilimidazole-*N*-biphenyltetrazole; AngII, angiotensin II; benzilimidazole *bis-N,N′*-biphenyltetrazole; ARBs, angiotensin receptor blockers; BV6(K^+)$_2$, 4-butyl-N,N0-bis[[20-2Htetrazol-5-yl)biphenyl-4-yl]methyl)imidazolium bromide; SEM, standard error of mean.

Table S1. Summary of effects of drug pre-treatment on inhibition of contraction responses to AngII, AngA, and PE. Abbreviations: ACC519T, benzilimidazole-*N*-biphenyl tetrazole; ACC519T(2), benzilimidazole *bis-N,N′*-biphenyl tetrazole; AngA, angiotensin A; AngII, angiotensin II; ARB, angiotensin II receptor blocker; BV6(K^+)$_2$, 4-butyl-N,N0-bis[[20-2Htetrazol-5-yl)biphenyl-4-yl]methyl)imidazolium bromide; ns, no significance; PE, phenylephrine.

ARB	AngII	AngA	PE
**ACC519T**	10^{-6} M to 10^{-40} M	10^{-6} M to 10^{-60} M	10^{-40} M
**ACC519T(2)**	10^{-6} M to 10^{-40} M	10^{-6} M to 10^{-60} M	10^{-12} M to 10^{-30} M
**BV6(K^+)$_2$**	10^{-6} M to 10^{-60} M	10^{-6} M to 10^{-60} M	10^{-12} M to 10^{-60} M
**Candesartan**	10^{-6} M to 10^{-60} M		10^{-12} M, 10^{-30} M, and 10^{-40} M
**Nirmatrelvir**	ns		ns
**Telmisartan**	10^{-6} M to 10^{-60} M		10^{-40} M
